# A retrospect and outlook on the neuroprotective effects of anesthetics in the era of endovascular therapy

**DOI:** 10.3389/fnins.2023.1140275

**Published:** 2023-03-28

**Authors:** Tianhao Zhang, Daling Deng, Shiqian Huang, Daan Fu, Tingting Wang, Feng Xu, Lulin Ma, Yuanyuan Ding, Kaixin Wang, Yafeng Wang, Wenjing Zhao, Xiangdong Chen

**Affiliations:** Department of Anaesthesiology, Union Hospital, Tongji Medical College, Huazhong University of Science and Technology, Wuhan, China

**Keywords:** neuroprotection, anesthetics, ischaemic stroke, endovascular procedures, therapy

## Abstract

Studies on the neuroprotective effects of anesthetics were carried out more than half a century ago. Subsequently, many cell and animal experiments attempted to verify the findings. However, in clinical trials, the neuroprotective effects of anesthetics were not observed. These contradictory results suggest a mismatch between basic research and clinical trials. The Stroke Therapy Academic Industry Roundtable X (STAIR) proposed that the emergence of endovascular thrombectomy (EVT) would provide a proper platform to verify the neuroprotective effects of anesthetics because the haemodynamics of patients undergoing EVT is very close to the ischaemia–reperfusion model in basic research. With the widespread use of EVT, it is necessary for us to re-examine the neuroprotective effects of anesthetics to guide the use of anesthetics during EVT because the choice of anesthesia is still based on team experience without definite guidelines. In this paper, we describe the research status of anesthesia in EVT and summarize the neuroprotective mechanisms of some anesthetics. Then, we focus on the contradictory results between clinical trials and basic research and discuss the causes. Finally, we provide an outlook on the neuroprotective effects of anesthetics in the era of endovascular therapy.

## Introduction

Stroke is the leading cause of disability and death worldwide. It can be classified into haemorrhagic stroke and ischaemic stroke, the latter of which is characterized by the sudden loss of blood flow to an area of the brain due to thrombosis or thromboembolism (Campbell et al., 2019). A nationwide community-based study showed that the incidence of acute ischaemic stroke (AIS) in all incident stroke cases was as high as 70%, and the high incidence and disability rates of AIS have seriously increased the socioeconomic and healthcare burdens (Gorelick, 2019; Wu S. et al., 2019). Nevertheless, only limited options for treatment are available at present.

Intravenous recombinant tissue plasminogen activator (IV-rtPA) was the only pharmacologic treatment approved by the United States Food and Drug Administration (FDA) until endovascular thrombectomy (EVT) emerged. IV-rtPA has played an integral role in treating AIS in recent decades. However, the multiple contraindications and narrow therapeutic window restrict the application of IV-rtPA (Patel et al., 2020). In addition, rtPA has a low recanalization rate (13–50%) in patients suffering from large vessel occlusion (LVO) because of the unresponsiveness of large thrombi to the enzyme (Saqqur et al., 2007).

Advances in interventional neuroradiology promoted the development of EVT. The publication of five clinical trials of EVT in 2015 with positive findings launched a new era in AIS treatment. EVT is beneficial to most patients with AIS caused by the occlusion of the proximal anterior circulation (Goyal et al., 2016). Compared with IV-rtPA, EVT has a broader application time window and can be used in patients with contraindications to thrombolysis or intracranial LVO.

Inevitably, EVT must be performed under anesthesia. Thus, anesthetics are more widely available to patients with AIS than ever before. The choice of anesthesia, however, is still based on team experience without definite guidelines. Recently, the option of general anesthesia (GA) and conscious sedation (CS) during EVT was discussed in many multicentre randomized controlled trials (RCTs) (Schonenberger et al., 2016; Simonsen et al., 2018; Goldhoorn et al., 2020; Maurice et al., 2022), which indicated that anesthetics may affect the outcomes of patients with EVT. Moreover, a retrospective study preliminarily showed that propofol anesthesia was related to improved functional independence compared with inhalational GA [odds ratio (OR) = 2.65; 95% confidence interval (CI), 1.14–6.22; *p* < 0.05] (Diprose et al., 2021). These effects may be attributed to the haemodynamic effects of anesthetic drugs or the neuroprotective properties of anesthetics (Simonsen et al., 2022). Whether anesthetics have neuroprotective effects will directly affect the selection of anesthesia for EVT treatment. However, different results on the neuroprotective effects of anesthetics have been shown in clinical trials and basic research.

In this paper, we describe the research status of anesthesia in EVT in Part 1 and summarize the mechanisms related to the neuroprotective effects of commonly used anesthetics in Part 2. Then, we focus on the contradictory results between clinical trials and basic research and discuss the causes of the heterogeneity in Part 3. Finally, we provide a brief outlook on the neuroprotective effects of anesthetics in the era of endovascular therapy.

## Anesthetics may affect the outcomes of EVT

With advances in stroke treatment, highly effective thrombectomy devices are being used more widely for patients with LVO (Wasselius et al., 2022). As a result, anesthetic drugs are more widely available to stroke patients than ever before. However, it remains unclear which type of anesthesia and what kind of anesthetic drug used in EVT are better for reducing postoperative complications and improving the prognosis.

### General anesthesia or conscious sedation

Thus far, the best anesthetic strategy during EVT is still a matter of debate. GA and CS are the two main anesthetic methods used in EVT. While allowing for immobility and airway control, GA can delay endovascular treatments and may be associated with hemodynamic instability. On the other hand, CS is faster and allows for neurologic assessment during a procedure, but thrombectomy can be less safe due to patient movement. As for which type of an anesthesia is better for the prognosis of patients, the views are constantly changing with the deepening of research. More than 10 years ago, a non-randomized retrospective study performed in 12 stroke centers in the United States demonstrated that GA was related to poorer neurological outcomes after 3 months (OR = 2.33; 95% CI, 1.63–3.44; *P* < 0.0001) (Abou-Chebl et al., 2010). In the same year, another study compared the safety and clinical outcomes between GA with intubation and CS in a non-intubated state (NIS). This study reported that a NIS was associated with lower infarct volume (OR = 0.25, *P* = 0.004) and better clinical outcomes (OR = 3.06, *P* = 0.042) (Jumaa et al., 2010). Although the same conclusion was drawn in the subsequent meta-analysis, the authors noted that patients receiving GA had higher average National Institute of Health Stroke Scale (NIHSS) scores in the 6 studies included (Brinjikji et al., 2015). This finding means that non-randomized retrospective studies have some methodological limitations (Talke et al., 2014; van den Berg et al., 2015). The stroke severity at baseline in the GA group and the CS group was inevitably imbalanced because the anesthetic protocol was decided by teams rather than by randomization (Albers et al., 2017). As a result, the severity of stroke in the GA group would be more severe than that in the CS group due to selection bias, which may have prevented drawing correct conclusions (Brinjikji et al., 2015; van den Berg et al., 2015).

Recently, a series of large-scale multicentre RCT studies on this topic have been carried out (Goyal et al., 2016), and different conclusions from previous retrospective studies have been drawn. The authors found that the functional outcomes of patients undergoing EVT after 3 months were similar in patients receiving GA and those receiving CS (relative risk, 0.91; 95% CI, 0.69–1.19), and even better recanalization was observed in the GA group (Goyal et al., 2016). In a meta-analysis including 3 RCTs [SIESTA (Schonenberger et al., 2016), ANSTROKE (Lowhagen et al., 2017), and GOLIATH (Simonsen et al., 2018)] and 368 patients with AIS in the anterior circulation, the application of GA during EVT was significantly associated with less disability on the 90th day (OR = 1.58; 95% CI, 1.09–2.29; *P* = 0.02) than the application of procedural sedation (Schonenberger et al., 2019). This may be because GA provides a more comfortable environment for the surgeon during EVT that will be safer and easier with a motionless patient (Chen et al., 2009). Recently, Simonsen et al. (2022) performed a mediator analysis to explore whether the better outcome in patients receiving GA was mediated by better recanalization and a higher reperfusion rate. Their meta-analysis also included 3 RCTs and 368 patients [SIESTA (Schonenberger et al., 2016), ANSTROKE (Lowhagen et al., 2017), and GOLIATH (Simonsen et al., 2018)]. The mediator analysis demonstrated that the indirect effect (i.e., better reperfusion) on outcome was small [risk difference (RD) = 0.03], and the direct effect of GA itself on outcome was much more significant (RD = 0.12). Moreover, they observed that even for non-reperfused patients, GA resulted in a better outcome than CS (Simonsen et al., 2022). This finding suggested the direct effects of GA, such as neuroprotection, as the source of a better outcome. An RCT in patients undergoing EVT where GA is induced by different anesthetic drugs could be valuable.

Before a definite conclusion is drawn, either GA or CS seems reasonable because GA and CS have their own advantages in EVT (summarized in Table 1). The American Heart Association and American Stroke Association guidelines advised selecting an anesthesia technique during EVT according to clinical characteristics, patient risk factors, and the technical performance of the procedure rather than a fixed anesthesia technique (Xie et al., 2016).

**TABLE 1:** Comparison of the advantages of general anesthesia and conscious sedation in EVT.

General anesthesia	Conscious sedation
Improve procedural conditions (Maurice et al., 2022)	Less haemodynamic instability (Davis et al., 2012)
Facilitate airway management	A shorter delay from arrival at the neurointerventional suite to groin puncture (Schonenberger et al., 2016)
Less pain, anxiety, and agitation and low aspiration risk (Emiru et al., 2014)	Fewer ventilation-associated complications (Takahashi et al., 2014)

### Different influences on haemodynamics

Improving collateral blood flow is a potential approach to protect the penumbra before recanalization (ENOS Trial Investigators, 2015; Savitz et al., 2019). Anesthetic agents can directly affect vessels and endogenous regulatory mechanisms (Nowak et al., 1984). Blood pressure reduction during EVT could impair collateral perfusion (Froehler et al., 2012). At present, there are no studies that have directly evaluated the haemodynamic effects of different anesthetic drugs on patients undergoing EVT. However, we can make some inferences from past research. Therefore, here, we summarize the findings of some past studies focusing on the effects of anesthetic drugs on cerebral haemodynamics.

(1) It has been debated for many years whether ketamine can be used as an anesthetic for neurologically compromised patients (Gregers et al., 2020). Early studies in the 1970s and 1980s reported that ketamine increased intracranial pressure (ICP), leading to a reduction in cerebral blood flow (CBF) and oxygen supply (Evans et al., 1971; List et al., 1972; Wyte et al., 1972; Nelson et al., 1980). However, subsequent studies found that when combined with propofol, ketamine (1.5, 3, and 5 mg.kg^–1^) could decrease ICP in patients with traumatic brain injury (Albanese et al., 1997). Subanaesthetic doses of ketamine increased regional cerebral blood flow (rCBF) in the frontal cortex (25.4% increase from baseline, *P* < 0.001) but did not change the regional metabolic rate of oxygen (rCMRO_2_) (Langsjo et al., 2003). A recent meta-analysis including 11 studies with a total of 334 patients showed that there was no evidence indicating that the application of ketamine worsened the cerebral condition (Gregers et al., 2020). It is currently thought that ketamine administration does not result in increased ICP when used as a part of a typical modern anesthesia protocol, and ketamine can be used safely in neurologically impaired patients (Himmelseher and Durieux, 2005; Slupe and Kirsch, 2018). However, no relevant studies have evaluated the safety of ketamine in EVT.

(2) Hypotension is a common side effect of propofol. As a result, the application of propofol in EVT necessitates higher requirements for blood pressure control since a drop of more than 40% in mean arterial blood pressure during EVT in GA is an independent risk factor for poor neurological outcomes (Lowhagen et al., 2015). Blood pressure is one of the determinants of CBF. In a study where positron emission tomography (PET) was used to quantify the effect of propofol on CBF and rCMRO_2_, propofol reduced rCBF and rCMRO_2_ to approximately 60% of the baseline at a concentration producing a bispectral index value of 40 (Slupe and Kirsch, 2018). Another similar study also showed a roughly equal reduction in rCMRO_2_ and rCBF (Himmelseher and Durieux, 2005), indicating that propofol could preserve the regional ratio between rCBF and rCMRO_2_. Thus, propofol has become an anesthetic in neurosurgical procedures (Gregers et al., 2020), but the haemodynamics of propofol in EVT should be further studied because haemodynamics do not change equally across the whole brain during EVT. Previous study findings may not apply to EVT.

(3) Volatile anesthetics such as sevoflurane and isoflurane have an intrinsic cerebral vasodilatory effect (Matta et al., 1999) that is related to the activation of adenosine triphosphate-sensitive K^+^ channels (Iida et al., 1998). Unlike propofol, sevoflurane and isoflurane at 1 minimum alveolar concentration (MAC) can increase CBF but decrease CMRO_2_ (Oshima et al., 2003), and this property may contribute partly to preventing postoperative ischaemic stroke. A retrospective cohort study that included 314,932 patients undergoing GA showed that volatile anesthesia was related to lower odds of postoperative ischaemic stroke compared with total intravenous anesthesia by propofol (Raub et al., 2021). However, in regard to application in EVT, the lesion and CBF autoregulation caused by volatile anesthetics should be considered. Autoregulation is a vasodilator reflex that maintains CBF within the physiological range under normal circumstances and helps build collateral blood supply around the ischaemic core after stroke (Hoffmann et al., 2016). It was reported that volatile anesthetics can impair autoregulation in rats and dogs (Archer et al., 2017; Esposito et al., 2020) and have similar effects in humans (Strebel et al., 1995; Goettel et al., 2016). It is necessary to carry out further research on the effects of volatile anesthetics.

### Neuroprotection in EVT

During AIS, a sudden decrease in blood flow to the brain area supplied by the blocked artery occurs, which is not uniform across the whole ischaemic area. The ischaemic core is the area in which < 20% of basal blood flow remains, and the penumbra is defined as the area where approximately 40% of the basal blood flow is maintained by collateral circulation (Zhao et al., 1997). The concept of neuroprotection involves preventing extraneuronal cell death by protecting the salvageable penumbral region around the ischaemic core after an ischaemic insult (Ginsberg, 2008). Although decades of failures have been experienced in clinical trials on neuroprotection, and none of the neuroprotective drugs have been approved for treatment, numerous studies are still ongoing.

**FIGURE 1:**
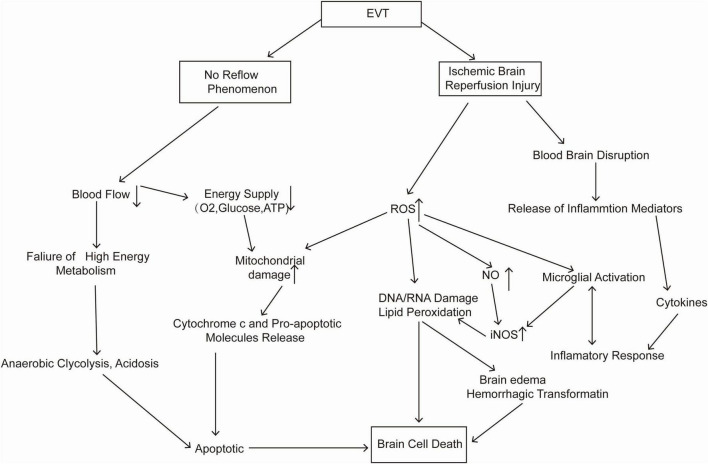
Pathological changes after endovascular thrombectomy (EVT).

Endovascular thrombectomy within 24 h of symptom onset could benefit patients with LVO (Berkhemer et al., 2015; Bracard et al., 2016; Nogueira et al., 2018). However, nearly 50% of patients may still undergo “futile recanalization” (Xu et al., 2020), which means that the recanalization of the occluded vessel fails to improve the neurological outcome (Nie et al., 2018). The no-reflow phenomenon after EVT may be one of the causes of futile recanalization. This phenomenon is defined as severe tissue hypoperfusion despite timely recanalization of an occluded artery, which may be due to abnormalities at the level of the microvasculature. Microvascular obstruction from endothelial cell swelling, pericyte contraction, luminal clogging with leukocytes and microthrombi can impede the reperfusion after EVT because EVT only clears blockages in large arteries (Nie et al., 2023). In clinical studies, the incidence of the no-reflow phenomenon after EVT has ranged from 25 to 38% (Ng et al., 2018; Rubiera et al., 2020; Ter Schiphorst et al., 2021). Another important cause of futile recanalization is cerebral ischaemia–reperfusion injury (Stoll and Nieswandt, 2019). During reperfusion, reactive oxygen species (ROS) are produced by the xanthine (XO) system, the NADPH oxidase (NOX) system, and the mitochondrial enzymatic system (Granger and Kvietys, 2015), leading to direct cellular damage and indirect damage, such as inflammation. Moreover, ROS can result in apoptosis and necrosis through lipid peroxidation and DNA/RNA damage (Mizuma et al., 2018). A more detailed mechanism is shown in Figure 1. Experimental studies showed that transient middle cerebral artery occlusion (3-hour occlusion and 3-hour reperfusion) in rats caused a larger infarct volume and blood–brain barrier disruption than permanent middle cerebral artery occlusion (6 h) (Yang and Betz, 1994). In clinical research, a similar ischaemia–reperfusion injury was indirectly observed in magnetic resonance imaging through a hyperintense acute reperfusion marker (Warach and Latour, 2004), suggesting that ischaemia–reperfusion injury also exists in humans. Therefore, neuroprotective drugs are particularly needed in EVT.

According to the Stroke Therapy Academic Industry Roundtable X (STAIR), in the current era of EVT, neuroprotective agents need to work synergistically with endovascular therapy to reduce ischaemia–reperfusion injury rather than work as monotherapies (Savitz et al., 2019). Perhaps the treatment of stroke is similar to precision surgery, which requires much cooperation. Neuroprotection in the new era should be verified on the basis of endovascular therapy. Therefore, many neuroprotective drugs that failed in clinical trials are currently being revisited (Yang et al., 2019). However, before that, anesthetics should be examined first in EVT, since anesthetics will be confounding factors in the validation of other drugs. For example, the neuroprotective effects of a tested drug might be masked if anesthetics also act on the same pathway.

## The neuroprotective properties of some anesthetic drugs in basic research

Over the decades, accumulating evidence has displayed the neuroprotective effects of anesthetic drugs involving multiple mechanisms and pathways. Here, we have selected several anesthetic drugs commonly used in clinical practice that have the neuroprotective potential for a brief discussion. We focus more on differences in the properties of different anesthetics and some studies with contradictory findings that may explain why these medicines “lose” their neuroprotective effects when used clinically.

### Ketamine

Ketamine is a phenyl cyclohexylamine derivative that consists of two optical enantiomers, (S)- and (R)- ketamine. The anesthetic properties of ketamine are mainly attributed to the direct inhibition of the N-methyl-D-aspartate receptors (NMDARs). Other lower-affinity pharmacological targets of ketamine include γ-aminobutyric acid (GABA) receptors, dopamine receptors, serotonin opioid receptors, cholinergic receptors, hyperpolarization-activated cyclic nucleotide-gated channels, and so on (Paoletti et al., 2013). The mechanisms of brain injury after stroke include excessive activation of NMDARs, an imbalance in intracellular and extracellular calcium concentrations, neuroinflammation, NO production, ROS production, apoptosis, and so on (Campbell et al., 2019). Blocking one of these mechanisms alone has only a limited effect. Studies on ketamine have found that its neuroprotective mechanism also involves multiple pathways and mechanisms.

**FIGURE 2:**
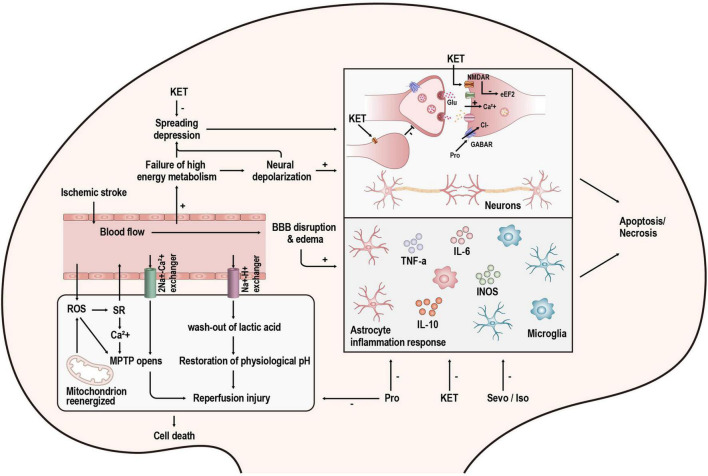
Overview of the mechanisms of injury after stroke and the targets of anesthetics. KET, ketamine; Pro, propofol; Sevo, sevoflurane; Iso, isoflurane.

N-methyl-D-aspartate receptors (NMDARs), ionotropic glutamatergic receptors, are permeable to calcium ions (Ca^2+^). These channels are blocked by magnesium at resting membrane potentials. However, when they are depolarized, the magnesium will be removed, and NMDAR conduction will be substantially higher (Nowak et al., 1984). In pathological conditions such as stroke, NMDAR overstimulation causes a series of Ca^2+^-dependent cascades of events (shown in Figure 2), which ultimately lead to neuronal demise. This process is excitotoxicity (Granzotto et al., 2022). Ketamine is a non-competitive inhibitor of NMDARs, and it can act on NMDARs in two ways. One is to block the open channel directly; the other is to act on the binding site outside the channel and indirectly affect NMDARs through an allosteric effect, reducing the number and frequency of NMDAR openings (Orser et al., 1997). In addition to the effects on NMDARs, ketamine has also been reported to affect glutamate release. A recent study showed that ketamine could reduce neuronal glutamate release by stimulating presynaptic adenosine A1 receptors (Lazarevic et al., 2021). However, other studies have demonstrated that ketamine application increases synaptic glutamate release (Abdallah et al., 2018; Lisek et al., 2017). This may be the result of differences in experimental design as well as in measurement methods.

Spreading depolarization (SD) is a kind of pathological wave that contributes to secondary lesions after stroke. The cumulative effect of many SDs is the same as a single persistent depolarization, leading to cell death and delayed lesions (Hartings et al., 2017). It has been proven that ketamine can suppress SD in acute brain injury (Carlson et al., 2018). In a retrospective international multicentre analysis, the administration of ketamine was associated with a reduction in spreading depolarizations (OR = 0.38; 95% CI, 0.18–0.79; *p* = 0.01) (Hertle et al., 2012). Moreover, Reinhart and Shuttleworth (2018) found that applying a lower concentration of ketamine (30 μM, brain slice) does not completely prevent SD but prevents its damaging consequences and retains the potential protective effect of SD. This finding is consistent with the study by Shu et al. (2012) in which they found that low-dose ketamine (25 mg.kg^–1^, intraperitoneal injection in rats) has a smaller infarct volume than high-dose ketamine (50 or 100 mg.kg^–1^, intraperitoneal injection in rats) in the treatment of stroke. However, there are also studies drawing contradictory conclusions. Some studies have shown that higher doses (60 and 90 mg.kg^–1^, intraperitoneal injection in rats) of ketamine improve neurological outcomes, but low doses do not (Reeker et al., 2000; Proescholdt et al., 2001). This difference may be associated with the different properties of R-ketamine and S-ketamine. Studies on S-ketamine tended to use high doses (Reeker et al., 2000; Proescholdt et al., 2001), whereas R-ketamine showed neuroprotective effects at low doses (Xiong et al., 2020). In an ongoing study in our laboratory, S-ketamine also initially showed a dose-dependent effect. The specific mechanism is being further studied.

Neuroinflammation and apoptosis are not only the result of the loss of ion homeostasis caused by NMDAR overactivation but also the cause of neuronal cell death. Ketamine has been proven to inhibit neuroinflammation (Tanaka et al., 2013; Liu et al., 2016; Wang et al., 2021) and apoptosis (Engelhard et al., 2003; Shu et al., 2012; Qi et al., 2020). Inflammatory factors and apoptosis-related molecules are dynamically changed in stroke patients. They not only change with time but also change drastically after recanalization in EVT. The timing and method of ketamine administration can significantly impact the outcome. In mice, applying ketamine by intraperitoneal injection immediately after ischaemia onset could not remarkably induce a significant change in infarct volume. However, injection immediately after the onset of ischaemia–reperfusion significantly reduced infarct volume (Xiao et al., 2012). Similarly, a preclinical study has shown that ketamine dramatically reduced infarct volume when combined with IV-rtPA. However, ketamine alone could not achieve this effect (Gakuba et al., 2011), which might be related to the upregulation of NMDARs after ischaemia–reperfusion (Sutcu et al., 2005). Many studies have confirmed that NMDARs are related to ischaemia–reperfusion injury, and antagonizing NMDARs can reduce ischaemia–reperfusion injury (Kaur et al., 2016; Xie et al., 2016; Singh et al., 2017). Using ketamine in combination with IV-rtPA may be a promising way to extend the time window of IV-rtPA. However, routine treatment with rtPA does not require the use of ketamine. In regard to EVT, anesthetic drugs are routinely used. If relevant studies could confirm that ketamine can reduce ischaemia–reperfusion injury and prolong the time window of EVT application, it will change the current situation where anesthesia during EVT is based on the experience and habits of anesthesiologists and lead to a better prognosis for patients.

### Propofol

Propofol is a widely used intravenous agent. Experimental studies have shown that propofol might protect the brain from ischaemic stroke (Bayona et al., 2004; Ulbrich et al., 2016; Wang et al., 2016). When propofol is used as an anesthetic drug for the induction and maintenance of anesthesia, it mainly acts by activating γ-aminobutyric acid (GABA_*A*_) receptors (Walsh, 2018). However, the function of GABA_*A*_ receptors in neuroprotection is complicated.

γ-aminobutyric acid (GABA) signaling has two forms. Tonic GABA signaling is a form of extrasynaptic GABA receptor-mediated inhibition. Reducing excessive GABA-mediated tonic inhibition promoted the recovery of motor function after stroke (Clarkson et al., 2010), indicating that excessive tonic inhibition is detrimental to the recovery of function. One of the interpretations was that the cortical hypometabolism caused by excessive astrocytic GABA would prevent functional recovery (Nam et al., 2020). At clinically relevant concentrations, propofol can affect extrasynaptic GABA receptors, although the effect is small (Wakita et al., 2013). Thus, propofol mainly affects the GABA receptors at the synapse rather than the extrasynaptic GABA receptors, which mediate a classic form of inhibition called phasic GABAergic inhibition. In the acute phase of stroke, enhancing phasic GABAergic inhibition can reduce excitotoxic neuron death (Lyden and Hedges, 1992; Green et al., 2000). Similarly, motor function can be improved when a GABA-positive allosteric modulator is used to enhance phasic GABAergic signaling during the repair phase (Hiu et al., 2016). However, due to the lack of direct evidence, further research on whether propofol exerts neuroprotective effects by enhancing phasic GABAergic signaling is needed.

Many studies have demonstrated the anti-apoptosis and anti-inflammation characteristics of propofol (Kotani et al., 2008; Fan et al., 2015; Peng et al., 2020; Qi et al., 2020). In addition to these classic effects, propofol has several other properties. It has a similar chemical structure to antioxidant substances such as vitamin E. It was reported that propofol could scavenge ROS, inhibit the generation of free radicals, and reduce lipid peroxidation to protect the brain from oxidative injury (Cheng et al., 2002; Kobayashi et al., 2008). Moreover, cell ferroptosis is one of the cell death processes correlated with overwhelming lipid peroxidation and cellular ROS. Recently, it was revealed that propofol may help attenuate ferroptosis in HT-22 cells treated with a ferroptosis activator (Erastin) (Xuan et al., 2022), providing a new therapeutic method to treat cerebral ischaemia. However, when used in cancer therapy, propofol appeared to enhance ferroptosis (Zhao and Chen, 2021; Zhao et al., 2022). Further study of the two opposing effects of propofol on ferroptosis is needed. Parthanatos is another form of programmed cell death induced by ROS. Zhong et al. (2018) found that propofol could inhibit parthanatos by impeding calcium release from the endoplasmic reticulum, ROS overproduction, and mitochondrial swelling.

As mentioned earlier, the excitotoxicity caused by glutamate and NMDARs has an essential impact on cerebral ischaemic injury. Propofol could inhibit NMDARs in some studies (Wu et al., 2018; Zhou et al., 2018), but the doses of propofol they used in their experiments exceeded clinically relevant concentrations. Another study on the effects of propofol on NMDAR-mediated calcium increase in neurons revealed that the overall effects of propofol were minor when the propofol concentration was at clinically relevant concentrations (Grasshoff and Gillessen, 2005). Therefore, the neuroprotective effect of propofol may be partly through the inhibition of NMDARs, but that is not the primary mechanism. Moreover, propofol may prevent excitotoxicity in other ways. Numerous studies have demonstrated that propofol can reduce glutamate concentrations during cerebral ischaemia by decreasing glutamate release (Ratnakumari and Hemmings, 1997; Lingamaneni et al., 2001) and increasing glutamate uptake (Cai et al., 2011; Gong et al., 2016). However, the glutamate concentration may not necessarily play a decisive role in the neuroprotective effect of propofol. Yano et al. (2000) found that propofol and Intralipid (a vehicle for propofol) could similarly reduce glutamate increase in CA1. In contrast, propofol, but not Intralipid, alleviated delayed CA1 neuron death when administered intracerebroventricularly in a transient global forebrain ischaemic model (Yano et al., 2000).

Hypothermia has been demonstrated to be an effective way to alleviate the damage caused by stroke (Gonzalez-Ibarra et al., 2011). When the ischaemic cascade is activated, therapeutic hypothermia can alleviate central nervous system hyperexcitability by reducing extracellular levels of excitatory neurotransmitters such as dopamine and glutamate. Hypothermia also protects the brain from ischaemic injury by reducing cerebral blood flow, oxygen and glucose consumption, and metabolic rate. The decrease in cerebral metabolic demands results in slower enzyme activity, allowing Adenosine triphosphate (ATP) stores to be preserved (Otto, 2015). When used in GA, propofol can induce heat redistribution from the core to the periphery by impairing thermoregulatory vasoconstriction and preventing shivering (Noguchi et al., 2002). However, it is difficult to quantify the extent to which the decrease in body temperature caused by propofol plays a role in its neuroprotective effect. This is because in basic experiments, we often use holding devices to ensure that the body temperature of the animals is constant and to prevent the neuroprotective effects of hypothermia from interfering with the experiment. In the context of temperature control, there are still basic research studies that confirm the neuroprotective effect of propofol (Fan et al., 2022).

The neuroprotective effect of propofol involves multiple mechanisms, but whether propofol can improve the long-term prognosis of stroke is uncertain. A study found that using propofol to treat cerebral ischaemia can significantly enhance the infarct volume and motor function on the third day after treatment. However, there was no difference in infarct volume on the 21st day in the propofol group compared with the control group (Bayona et al., 2004). In addition, in a preclinical trial of propofol combined with IV-rtPA, propofol failed to reduce infarct size after thrombolysis (Gakuba et al., 2011). Some clinical studies did not support the neuroprotective effect of propofol (as shown in Table 1). The reasons for this difference will be discussed in detail in the second part.

### Sevoflurane and isoflurane

Sevoflurane and isoflurane are both commonly used volatile anesthetics for the induction and maintenance of GA. The targets of these inhaled anesthetics include but are not limited to GABARs, NMDARs, and TWIK-related K^+^ channels (TREK-1) (Orser et al., 2019). As mentioned before, NMDARs play a vital role in excitotoxicity. Although volatile anesthetics can protect against excitotoxicity partly by inhibiting NMDARs, the efficiency of volatile anesthetics is less than selective NMDAR antagonism (Kudo et al., 2001). Thus, the neuroprotective effect of volatile anesthetics partly contributes to NMDAR inhibition, but this is not the main mechanism.

Some existing studies have indicated that sevoflurane and isoflurane can reduce ischaemia and ischaemia–reperfusion injury by affecting inflammatory and apoptotic processes (Bedirli et al., 2012; Hwang et al., 2017; Zhang and Zhang, 2018; Yang et al., 2022). A recent review of the neuroprotective mechanisms of sevoflurane and isoflurane specifically summarized how they affect classic inflammatory and apoptotic pathways (Neag et al., 2020). However, not all reports about inhaled anesthetics are positive (Zhang et al., 2016; Wu et al., 2020). Orset et al. (2007) developed a mouse model of thromboembolic stroke that is closer to the physiological situation than traditional stroke models. Then, Gakuba et al. (2011) used this model to assess the different effects of the combination of anesthetics and IV-rtPA on the infarct volume. Unexpectedly, isoflurane and propofol failed to enhance the benefits brought by rtPA-induced thrombolysis (Gakuba et al., 2011). Moreover, sevoflurane applied in different models can even have the opposite effect. When used in rats that were subjected to brain hypoxia-ischaemia, sevoflurane could protect the brain by inhibiting apoptosis (Ren et al., 2014). However, sevoflurane showed neurotoxicity and tended to exacerbate apoptosis when rat pups were exposed to it for as long as 4 h (Shan et al., 2018).

Therefore, it seems that simply evaluating whether a drug is neuroprotective is unscientific. The protective effect is based on a specific environment, and the application of the same medication to different subjects at different doses can even produce opposite effects. For example, the effects of anesthetic drugs on NMDARs, GABARs, or some other receptors may be detrimental in some patients but may reduce excitotoxicity in patients experiencing cerebral ischaemia. The narrow concept of neuroprotection is based on the condition of ischaemia, and it is a process that reduces brain injury after the onset of stroke (Ginsberg, 2008). Therefore, our clinical research on the neuroprotective effects of anesthetics should be precisely linked to stroke. However, many clinical studies in the past have used other diseases and surgeries to study the neuroprotective effects of anesthetics (summarized in Table 2). Past studies may not accurately evaluate the neuroprotective effect of anesthetic drugs.

**TABLE 2:** Clinical studies on the neuroprotective effects of propofol, ketamine, sevoflurane and isoflurane.

References	Research type	Comparison of drug treatment	Experimental subjects	Outcomes
Bhutta et al., 2012	RCT (*n* = 24)	Ketamine (2 mg.kg^–1^) vs. placebo (saline)	Infants undergoing cardiopulmonary surgery	No evidence for neuroprotection or neurotoxicity.
Loo et al., 2012	RCT (*n* = 46)	Ketamine (0.5 mg.kg^–1^) or placebo (saline)	Patients undergoing electroconvulsive therapy	Slight improvement in the first week of treatment.
Nagels et al., 2004	RCT (*n* = 106)	S (+)-ketamine (2.5 mg.kg^–1^) vs. remifentanil	Patients undergoing open-heart surgery	No greater neuroprotective effects than with remifentanil.
Hudetz et al., 2009	RCT (*n* = 26)	Ketamine (0.5 mg.kg^–1^) vs. placebo (saline)	Patients undergoing open-heart surgery	Ketamine attenuated POCD 1 week after cardiac surgery.
Guo et al., 2019	RCT (*n* = 60)	Propofol (1.2 μg.ml^–1^, TCI, plasma target concentration) vs. 0.5–2% sevoflurane	Patients undergoing aneurysm clipping	Propofol may protect the brain from oxidative stress injury up to 7 days.
Tanguy et al., 2012	RCT (*n* = 59)	Propofol (depending on the procedure requirements) vs. midazolam (depending on the procedure requirements)	Patients with severe traumatic brain injury	Results did not support a difference between propofol and midazolam for sedation in traumatic brain injury.
Kanbak et al., 2004	RCT (*n* = 20)	Isoflurane (1 to 1.5% until CPB and 0.5 to 1% during CPB) vs. propofol (6 mg.kg ^–1^.h^–1^ until CPB and 3 mg.kg^–1^.h^–1^ during CPB)	Patients undergoing coronary artery bypass grafting	Propofol appeared to offer no advantage over isoflurane for cerebral protection during cardiopulmonary bypass.
Schoen et al., 2011	RCT (*n* = 128)	Propofol (3–5 mg.kg^–1^.h^–1^) vs. sevoflurane (0.6-1MAC)	Patients undergoing on-pump cardiac surgery	Sevoflurane-based anesthesia was associated with better short-term postoperative cognitive performance than propofol.
Mahajan et al., 2014	RCT (*n* = 66)	Propofol (attain a burst suppression ratio of 75 ± 5% in bispectral index monitoring) vs. placebo (saline)	Patients undergoing temporary clipping during intracranial aneurysm surgery	Propofol did not offer any neuroprotective effects on improving postoperative cognition.
Roach et al., 1999	RCT (*n* = 225)	Propofol (computer-assisted continuous infusion titrated to achieve EEG burst suppression) and sufentanil (5 μg.kg^–1^) vs. sufentanil (5 μg.kg^–1^)	Patients undergoing cardiac valve replacement	Propofol did not significantly reduce the incidence or severity of neurologic or neuropsychologic dysfunction.
Wu B. et al., 2019	RCT (*n* = 80)	Propofol (depending on the procedure requirements) vs. dexmedetomidine (depending on the procedure requirements)	Patients undergoing endovascular therapy	This study did not show any difference between propofol and dexmedetomidine in good outcomes or in-hospital mortality.
Yoon et al., 2020	RCT (*n* = 152)	Sevoflurane vs. no intervention	Patients with moyamoya disease undergoing revascularization surgery	Sevoflurane postconditioning did not reduce the incidence of SCH after revascularization surgery in patients with moyamoya disease.
Dabrowski et al., 2012	Observational study (*n* = 128)	Sevoflurane vs. isoflurane vs. control	Patients undergoing coronary artery bypass graft surgery	Isoflurane and sevoflurane reduced brain injury markers such as plasma matrix metalloproteinase-9 and glial fibrillary acidic protein.

POCD, postoperative cognitive dysfunction; CPB, cardiopulmonary bypass.

## The considerable gap between clinical trials and basic research

Patients undergoing endovascular therapy need GA or CS to undergo the procedure. However, there are few guidelines to help in the selection of anesthetic drugs. Although the findings from many basic research studies support the neuroprotective effects of anesthetics (Sanders et al., 2005), the results are ambiguous when evaluating anesthetic neuroprotective effects in clinical trials. Here, two authors independently searched PubMed and Medline for randomized controlled trials published between 1 January 1995 and 1 September 2021, using the permutation and combination of the keyword terms “neuroprotective,” “neuroprotection,” “ischaemia,” “ketamine,” “propofol,” “sevoflurane,” and “isoflurane”; excluded the studies that were not relevant to the theme of this paper after discussion; and finally summarized the results in Table 2. We can see in Table 2 that the conclusions of these clinical trials are not unified, and some are even contradictory. Here, we discuss why there is a considerable gap between clinical trials and basic research.

### Defects in basic research

According to the STAIR criteria (Fisher et al., 2009), a large number of studies on neuroprotection seem to exhibit low methodological quality. Here, we summarize some common defects in research on the neuroprotective effects of anesthetics.

(1) Some basic research focuses more on infarct volume (Shu et al., 2012; Xiong et al., 2020) than on subsequent outcome several months later, which is commonly evaluated through the modified Rankin Scale (mRS) in clinical trials (Saver et al., 2021). The latest STAIR trial advised that the main endpoints should include not only infarct volume but also behavioral outcomes, gray versus white matter protection, and the potential negative effects of the agent tested (Savitz et al., 2019).

(2) Another problem is the incompatibility between the doses of medicine used in basic research and those used in clinical practice. Due to receptor affinity, some drugs that show significant neuroprotective effects at concentrations higher than clinically applied often fail to improve patient prognosis after entering clinical studies (Muir, 2006; Morgan et al., 2012), and their clinical application value is limited.

(3) Basic research studies pay more attention to whether an anesthetic drug has a neuroprotective effect, so they tend to determine the timing when the phenomenon is most obvious through preliminary experiments and then proceed from there (Zhou et al., 2013; Yang et al., 2018). However, clinical practice has more demand for the time window of drug application since patients suffering from stroke have a variable duration of ischaemia. If the time window of a drug is very narrow or the time of administration and the method of administration is unrealistic (Saver et al., 2021), its clinical significance is still limited even if a positive result is obtained.

(4) Transient middle cerebral occlusion (tMCAO) is the most widely used model of stroke and has advantages in the study of reperfusion injury. With the continuous development of endovascular therapy, it is increasingly important to research how to reduce ischaemia/reperfusion injury and promote prognosis. However, there is still a large number of patients without vessel recanalization (Yoshimura et al., 2014), which is closer to permanent middle cerebral occlusion (pMCAO). When we evaluate the neuroprotective effects of drugs, pMCAO should also be taken into consideration (McBride and Zhang, 2017).

(5) Sex and age have long been neglected factors. A meta-analysis including 80 publications compared the neuroprotective effects of anesthetics in animals of different sexes and aged animals. It showed neuroprotective effects in female and aged animals (Archer et al., 2017). Although it was based on a *post hoc* analysis and a small number of studies, this meta-analysis raised a thought-provoking question: Are normal male animals appropriate animals in which to simulate human stroke?

(6) Clinical trials mostly test neuroprotectants in active, awake patients. In five large clinical trials of neuroprotectants involving 9,560 patients, only 664 had suffered night-time strokes (Esposito et al., 2020). However, rodent tests are always performed during the day, when they are inactive. The opposite circadian rhythm of rodents to that of humans impacts the effectiveness of neuroprotectants, which may be one reason for translational failure (Esposito et al., 2020; Boltze et al., 2021). Some moderate-quality studies have shown that anesthetic drugs affect circadian rhythms (Orts-Sebastian et al., 2019; Imai et al., 2020; Wang et al., 2020). Therefore, the influence of circadian rhythm must be considered for translational studies on anesthetic neuroprotection.

### The transient effects of anesthetic drugs

Common anesthetic drugs such as propofol, ketamine, and volatile anesthetics all have a short half-life in humans (Freiermuth et al., 2016; Peltoniemi et al., 2016; Sahinovic et al., 2018), which is an advantage in fast recovery after anesthesia. However, in regard to neuroprotection, the transient effects of anesthetic drugs may become a disadvantage because some injurious factors can last for a long time. For example, the elevation of excitatory amino acid (EAA) concentrations in MCAO lasts only 1–2 h (Takagi et al., 1993; Baker et al., 1995); however, in humans with AIS, glutamine increase may persist for 24 h or longer (Bullock et al., 1995; Davalos et al., 1997). Moreover, microglial cells, a type of immune cell in the brain, peak in activity 2–3 days after injury (Barthels and Das, 2020), and they can release variable inflammatory factors that lead to secondary injury around the ischaemic core (Yenari et al., 2010). As a result, the injurious factors are still in effect after the neuroprotective effects of anesthetics have passed. In addition, the compensatory function of patients is established within several months after suffering a stroke. This may be the reason why the neuroprotective effect of anesthetic drugs is not significant when we evaluate the recovery of neurological function of patients after several months in clinical studies. In the future, we may be able to introduce some therapies suitable for long-term use to restrict damage-causing factors. Electroacupuncture may be an option. Electroacupuncture, an extension of traditional acupuncture, is used as a complementary treatment with minimal side effects (Wei et al., 2016). Studies have shown that electroacupuncture can attenuate inflammation after ischaemic stroke by inhibiting the activation of microglia (Liu et al., 2020), improving cerebral blood flow, and alleviating neurological deficits (Zheng et al., 2016). As anesthetic drugs are not suitable for prolonged use after EVT, electroacupuncture can be used as an adjunctive technique to help reduce ischaemia–reperfusion injury after recanalization and to promote functional recovery. However, the reporting quality of randomized controlled trials on electroacupuncture for stroke is generally moderate, and further improvement is needed (Wei et al., 2016).

### Heterogeneity in experimental subjects

In MCAO, a filament is sent into the middle cerebral artery from the internal or external carotid arteries to mimic stroke, and it allows reperfusion through the withdrawal of the filament (Smith et al., 2015). This kind of reperfusion is different from the pathophysiology of thrombolysis in human stroke because the blood flow is restored promptly. Compared with thrombolysis, MCAO more closely simulates the clinical situation of mechanical thrombectomy (Sommer, 2017). However, past clinical studies on the neuroprotective effects of anesthetic agents were based neither on patients undergoing thrombolysis nor on those undergoing mechanical thrombectomy. As shown in Table 2, a large part of the past clinical research is based on other operations or diseases that may cause cerebral ischaemia, such as heart surgery and intracranial aneurysm surgery. These studies are not sufficiently convincing to evaluate whether anesthetic drugs have neuroprotective effects because most of these surgeries cause only transient ischaemia and postoperative cognitive dysfunction, which cannot cause large areas of brain tissue necroptosis such as stroke.

In addition, stroke is a heterogeneous disease with diverse additive risk factors (Caprio and Sorond, 2019). Although strict inclusion and exclusion criteria and grouping can reduce the effect of patient heterogeneity, the heterogeneity of patients in clinical studies is still more significant than that in animal models. The emergence of EVT provides a good translational platform for drugs that exhibit neuroprotective effects in the MCAO model. Among patients undergoing EVT, we were able to screen out patients with similar proximal intracranial artery occlusion by Computed Tomography (CT) and angiography, in which the heterogeneity of haemodynamics will be smaller and the haemodynamic changes will be much closer to those of MCAO.

There are already large multicentre, double-blind, randomized controlled trials of neuroprotective drugs in EVT patients (Hill et al., 2020). In the future, more rigorous basic research and clinical trials based on EVT will more rationally evaluate the neuroprotective effects of anesthetics. Regardless of the outcome, this research will provide more conclusive answers to decades-old questions on the neuroprotective effects of anesthetics.

## Conclusion

Anesthetics have great potential in neuroprotection, involving various mechanisms such as excitotoxicity, SD, inflammation, apoptosis, and ischaemia–reperfusion injury, but this has not been clearly observed in previous clinical trials due to the mismatch between basic research and clinical trials. The emergence of EVT has brought new hope to the study of the neuroprotective effects of anesthetics that once had been shelved. EVT might become a bridge connecting basic and clinical research. Anesthetics have long been confounding factors in translational stroke research. With an increasing number of neuroprotective techniques coming into clinical trials (Baker et al., 1995; Davalos et al., 1997; Sahinovic et al., 2018), it is necessary to determine the effects of anesthetics during EVT, and anesthetists also need a definitive study to guide clinical anesthetic administration.

## Author contributions

TZ, DD, SH, and DF contributed substantially to the article concept and manuscript writing. TZ, SH, FX, LM, YW, YD, and KW retrieved the literature and reviewed the manuscript. XC and TW revised and approved the final version before submission. All authors have participated actively in the study and have read and approved the final manuscript.
